# Influenza and Cholera

**Published:** 1848-02

**Authors:** 


### THE MEDICAL EXAMINER.

### PHILADELPHIA, FEBRUARY, 1848.

### INFLUENZA AND CHOLERA.

According to late accounts from Europe, the influenza was prevailing to an extraordinary extent in London, Paris, and most of the other great cities, and, as a similar epidemic, in some places, preceded the occurrence of Asiatic cholera, it seems to be generally apprehended by the practitioners of Britain and France that the latter disease will soon reach them. The journals are much occupied with discussions as to the pathology and treatment of the disease, and the proper preventive measures. In the London Medical Gazette of the 17th of December last, it is announced that "Dr. Arthur Farre and Mr. Toynbee have been appointed by government to inspect the various work-houses and infirmaries in the metropolitan parishes, to determine the amount of accommodation for pauper patients, in the event of Asiatic cholera breaking out in this city," (London.)

